# Enhancement of Drought-Stress Tolerance of *Brassica oleracea* var. *italica* L. by Newly Isolated *Variovorax* sp. YNA59

**DOI:** 10.4014/jmb.2006.06010

**Published:** 2020-08-13

**Authors:** Yu-Na Kim, Muhammad Aaqil Khan, Sang-Mo Kang, Muhammad Hamayun, In-Jung Lee

**Affiliations:** 1 School of Applied Biosciences, Kyungpook National University, Daegu 41566, Republic of Korea; 2 Institute of Agricultural Science and Technology, Kyungpook National University, Daegu 41566, Republic of Korea; 3 Department of Botany, Abdul Wali Khan University, Mardan, Pakistan

**Keywords:** *Variovorax* sp. YNA59, drought stress, phytohormones, antioxidant activity, broccoli

## Abstract

Drought is a major abiotic factor and has drastically reduced crop yield globally, thus damaging the agricultural industry. Drought stress decreases crop productivity by negatively affecting crop morphological, physiological, and biochemical factors. The use of drought tolerant bacteria improves agricultural productivity by counteracting the negative effects of drought stress on crops. In this study, we isolated bacteria from the rhizosphere of broccoli field located in Daehaw-myeon, Republic of Korea. Sixty bacterial isolates were screened for their growth-promoting capacity, in vitro abscisic acid (ABA), and sugar production activities. Among these, bacterial isolates YNA59 was selected based on their plant growth-promoting bacteria traits, ABA, and sugar production activities. Isolate YNA59 highly tolerated oxidative stress, including hydrogen peroxide (H_2_O_2_) and produces superoxide dismutase (SOD), catalase (CAT), and ascorbate peroxidase (APX) activities in the culture broth. YNA59 treatment on broccoli significantly enhanced plant growth attributes, chlorophyll content, and moisture content under drought stress conditions. Under drought stress, the endogenous levels of ABA, jasmonic acid (JA), and salicylic acid (SA) increased; however, inoculation of YNA59 markedly reduced ABA (877 ± 22 ng/g) and JA (169.36 ± 20.74 ng/g) content, while it enhanced SA levels (176.55 ± 9.58 ng/g). Antioxidant analysis showed that the bacterial isolate YNA59 inoculated into broccoli plants contained significantly higher levels of SOD, CAT, and APX, with a decrease in GPX levels. The bacterial isolate YNA59 was therefore identified as *Variovorax* sp. YNA59. Our current findings suggest that newly isolated drought tolerant rhizospheric *Variovorax* sp. YNA59 is a useful stress-evading rhizobacterium that improved droughtstress tolerance of broccoli and could be used as a bio-fertilizer under drought conditions.

## Introduction

Plants are constantly exposed and challenged by various environmental stresses throughout their life cycle. Among these environmental stresses, drought stress causes significant losses to agricultural productivity [1], as it reduces germination rates, seedling height, leaf area, and crop growth [2-4]. In China, drought stress reduced more than 30% of corn yield [5], while reduction in maize yield (40%), and wheat (21%) were recorded during 40%of water reduction [6]. Similarly, a 34%–68% yield reduction in cowpea depending on the timing of drought stress [7]. Drought stress leads to the generation of reactive oxygen species (ROS) such as superoxide anion (O^2−^), hydrogen peroxide (H_2_O_2_), and hydroxyl radical (HO) and cause oxidative damage to cell components, lipid and protein peroxidation, and enzyme inhibition eventually leading to cell death [8, 9]. Drought tolerance may be improved through various methods, including plant breeding, chemical fertilizer, tissue culture, and genetic engineering, which are time consuming, costly, and have adverse effects on the environment.

The use of plant growth-promoting rhizospheric bacteria (PGPR) is an ecofriendly approach for improving agricultural productivity by easing the negative effect of drought stress on economically important plant species worldwide. PGPR can improve drought tolerance by regulating phytohormone production, biological nitrogen fixation, the production of siderophores, hydrogen cyanide (HCN), ammonia, and phosphate solubilization [10, 11]. The secondary metabolite secreted by PGPR strengthens plant resistance to various stressors and promotes growth and yield attributes of treated plants grown under drought conditions. Previously, several researchers have reported the use of PGPR to ease drought stress in plants such as *Lavandula dentate* [12], soybean [13], maize [14], and basil plants [15].

*Brassica oleracea* var. *italica* L. belongs to the family Brassicaceae, and is recognized for its health benefits, provision of a good source of bioactive phytochemicals, and consumed as vegetable worldwide [16]. The chemical composition of broccoli makes it popular for human diet because it contains secondary metabolites (glucosinolates and flavonoids), protein, carbohydrates, minerals, and vitamins. According to previous research, drought stress severely affects broccoli growth, biomass, and yield [17, 18]. The application of bio-stimulants and nitric oxide also enhances drought stress tolerance in broccoli [19, 20], while the inoculation of PGPR enhanced growth attributes, and biomass under normal condition [21-23]. There is no previous report regarding the application of PGPR to broccoli under drought stress. In this study, we evaluated the effect of PGPR on the physiological traits, endogenous phytohormones, and antioxidant levels of broccoli grown under PEG induced drought stress conditions.

## Materials and Methods

### General Procedure

Bacteria were isolated from the rhizosphere of broccoli field in Daehaw-myeon, Gangwon-do, Republic of Korea (latitude 37°32' 20.3"N, longitude 128°27'05.8"E). One g of soil sample was suspended in 9 ml of sterilized 0.85% NaCl and the suspensions were serially diluted up to 10^−5^ as previously described [24, 25]. All isolates were screened for different plant growth-promoting traits. Orange media test was used to check the catalase production ability of the isolates. The composition of orange media was 100 ppm reactive orange16, lysogeny broth (LB), and agar 1.8% [26]. Congo red assay was used to confirm the formation of bacterial exopolysaccharides (EPS) [27]. Assay plates were prepared with LB broth (25 g/l), sucrose (5%), agar (2%), and Congo red (0.8 g/l), autoclaved, inoculated, and incubated for 5 days at 37°C. Six concentrations of PEG 6000 (0, 5, 10, 15, 20, and 25%) were prepared and 0.1% of culture aliquot was inoculated into 5 ml of sterilized LB broth and incubated in a shaking incubator at 30°C. The optimal density was measured using T60 UV-Vis spectrophotometer (PG Instruments Ltd., UK) at 600 nm. On the basis of screening results, bacterial isolate YNA59 was selected for further detailed study.

### In vitro Quantification of Abscisic Acid and Sugar Content of Bacterial Isolate YNA59

Bacterial isolate YNA59 was grown on LB media for 3 days, centrifuged (5000 ×g; 15 min) culture filtrate was analyzed for ABA and sugar contents. For ABA measurement, following the method previously published by Khan *et al* . [25, 28]. The concentration of ABA content was calculated by comparing with the known standard using the gas chromatography/mass spectrometry with selected ion monitoring (GC/MS SIM)

For sugar content, the method described by Kang *et al* . [29] was followed. 1.5 ml of isolate YNA59 culture, which was incubated for 5 days at 30°C was centrifuged (8000 rpm; 10 min), filtered through Sep pack C18 cartridge and 0.45 μm Nylon-66 syringe filters and analyzed through high-performance liquid chromatographic (HPLC) technique.

### Stress Media Test of YNA59

Isolate YNA59 was tested for tolerance to multiple oxidative stresses. Oxidative stress test was conducted by following the method of Park *et al* . [30] by supplementing LB agar media with different concentrations of hydrogen peroxide (0 mM—2 mM). For superoxide dismutase (SOD) measurement, SOD Assay Kit-WST (Dojindo Co. Ltd, Japan) was used, while the catalase activity was determined using the Amplex Red Catalase Assay Kit (Molecular Probes, Thermo Fisher, USA). Ascorbate peroxidase (APX) was determined according to the method described by Khan *et al* . [31] by measuring the absorbance at 290 nm using a T60 UV-Vis Spectrophotometer.

### Growth Condition and Treatments

Broccoli seeds were purchased from Asia Seed Korea Ltd, Seoul, Republic of Korea and sown in trays filled with autoclaved horticultural soil (which contained coco peat 51.5%, peat moss 10%, vermiculite 13%, perlite 15%, zeolite 10%, humic acid, 0.1% fertilizer, and 0.4% fungus-free bio-soil, Gyeonggi-do, Shinsung Mineral Co., Ltd, Republic of Korea). After 2 weeks of germination, seedlings were transferred to pots (10 × 10) and were grown in a growth chamber at a temperature of 22°C ± 0.5°C, 65% relative humidity, and light intensity of 200 μmol m^−2^ s^−1^ under long-day conditions (16 h of day time and 8 h of night time). The experimental design includes (a) W.C: Control- well watered (200 ml water per week) (b) V.C: Bacterial treated (200 ml cell suspension of isolate YNA59/week), (c) W.D: drought stress 50 ml/week (d) V.D: drought stress treated with 50 ml YNA59/week. To test plant protection activity of YNA59 under drought stress, 50 ml YNA59 (4.0 × 10^8^ cfu/ml) were inoculated via the soil drench method, while distilled water was used for control plants for 2 weeks. After stress completion, growth attributes and biomass were determined, plants were immediately harvested in liquid nitrogen and stored at -80°C until further biochemical analyses. Before the harvest, chlorophyll contents were measured using the chlorophyll meter 300 (ADC BioScientific Ltd., UK).

### Quantification of Endogenous Phytohormones

The endogenous phytohormones of plant samples were analyzed and quantified in a controlled environment. Endogenous ABA measurement was performed following an established protocol [32, 33]. Briefly, 0.1 g freeze-dried plant samples were extracted with isopropanol: acetic acid (95:5) filtered and added 50 ng/ml of ABA standard. The extracts were dried with N2 gas and methylated using diazomethane for ABA detection in a GC/MS-SIM. Plant endogenous JA was quantified following the method of [24]. Alternatively, SA was extracted from freeze-dried broccoli samples following the protocol [34].

### In-vivo Visualization of ROS in Broccoli Leaves

To visualize ROS damage, 3,3'-Diaminobenzidine (DAB) staining was conducted by following the protocol of [35]. The first and second leaves of broccoli, (highly damaged by drought stress) were incubated in 1% solution of DAB (Sigma Aldrich) and fully covered with broccoli leaves until the light and dark brown spots showed up. After incubation, leaves were bleached by boiling a decolorizing solution (acetic acid: glycerol: 96% ethanol = 1:1:3) to clearly visualize blue and brown spots, respectively, and after which they were photographed. All solutions were prepared in potassium phosphate buffer at pH 7.4.

### Quantification of Total Protein and Antioxidants

For protein analysis, frozen fresh plant tissues were ground with ice-cold pestle and mortar, and then added to a solution of 50 mM phosphate buffered saline, 0.1% polyvinylpyrrolidone (PVP), and 1 mM ethylene diamine (EDTA). The homogenate was centrifuged at 10000 ×*g* for 10 min at 4°C. The supernatant was immediately collected and used for protein and antioxidant enzyme quantification. For protein contents; Bradford [36] method was used in accordance with the BSA as a standard. Superoxide dismutase (SOD) was measured using SOD Assay Kit-WST, while catalase was determined. Ascorbate peroxidase (APX) and guaiacol peroxidase (GPX) was determined in accordance with the method described by Khan *et al* . [31] and Chaoui *et al* . [37] by measuring the absorbance at 290 nm and 470 nm using a T60 UV-Vis spectrophotometer.

### Identification of Bacterial Isolate YNA59

Isolate YNA59 was incubated on cover glasses for 5 days. The cover glass pieces covered with a film of YNA59 were fixed with 2.5% glutaraldehyde for 2 h and washed with 0.1 M sodium acetate buffer (pH 7.3). The glass pieces were dehydrated in ethanol (20%, 50%, 70%, 80%, 90%, and 100%) gradually. Before acquiring the field emission scanning electron microscope (FE-SEM) (Hitachi SU8220, Japan), the samples were sputter-coated with gold using an ion-sputtering device (JFC-110E, EC&G, USA), and examined with FE-SEM [38]. Based on their best performance during the screening experiments, isolate YNA59 was selected for further experimentation and identification. For molecular identification, the method of Sambrook and Russell [39] was used for genomic DNA extraction, 16S rRNA-specific primers were used and amplified according to the protocol of Khan *et al* . [31]. The NCBI BLAST program was used to determine the homology of different nucleotide sequences of selected isolates, and MEGA 6.1 software was then used for phylogenetic analysis [40].

### Statistical analysis

The results were statistically evaluated by analysis of variance using SAS 9.4 software. All analyses were repeated thrice with 14 plants per replicate. Duncan’s multiple range tests were used to determine 95% confidence level.

## Results

### Isolation and screening bio-assay of bacterial isolates

**Fig. 1:**
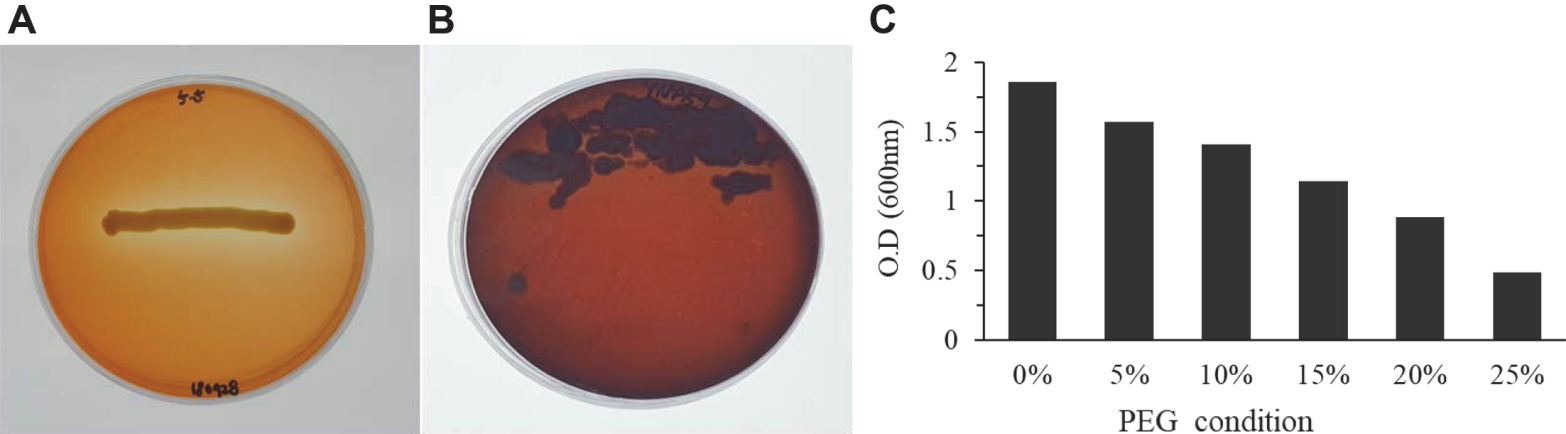
Catalase and exopolysaccharide (EPS) activity as displayed on orange media and Congo red medium. (**A**) Shows the capability of catalase production, (**B**) EPS production, and (**C**) growth of isolate YNA59 at different concentrations of PEG6000.

A total of 60 bacterial isolates were collected from the rhizospheric soil of broccoli plants. The isolated bacteria were screened for different PGP traits, like the catalase assay, EPS formation, and PEG tolerance (Fig. S1. Orange media results showed that 18 bacterial isolates showed catalase activity (Fig. 1A and Fig. S1A). The bacterial isolates were further screened on Congo red assay, and 4 isolates showed positive results (Fig. 1B and Fig. S1B). The selected 4 isolates were then screened at different concentrations of PEG (0%, 5%, 10%, 15%, 20%, and 25%), and one isolate YNA59 showed the highest PEG induced drought tolerance (Fig. 1 and Fig. S1C). Therefore, isolate YNA59 was selected for further investigations (Fig. 1).

### *In vitro* Abscisic Acid and Sugar Quantification of Isolate YNA59

**Fig. 2:**
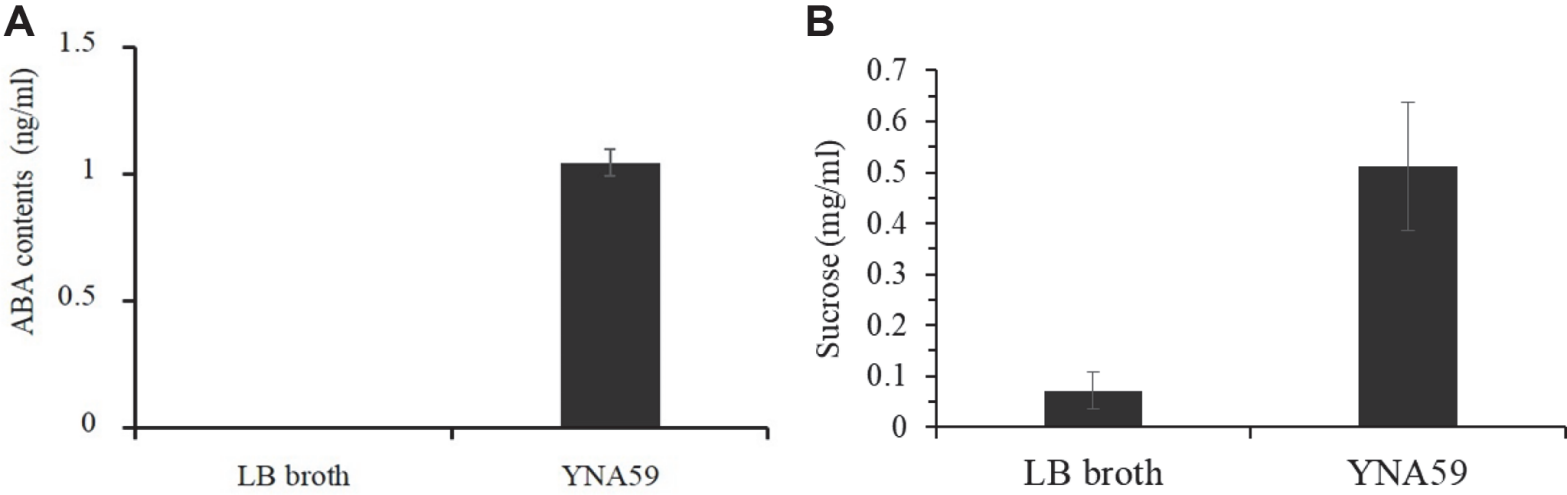
Characteristics of bacterial isolates. (**A**) Quantification of abscisic acid (ABA) observed in culture broth (LB broth) of YNA59 and (**B**) Quantification of sucrose content in LB of YNA59. Each data point is the mean of three replicates. Error bars represent standard errors. The bars represented by different letters are significantly different from each other as evaluated by Duncan’s multiple range tests (DMRTs).

The culture filtrate of isolate YNA59 was quantified for ABA by using GC/MS and sugar content through HPLC. ABA results showed that isolate YNA59 produced a significant amount of ABA (1.04 ± 0.05 ng/ml)(Fig. 2A). Sugar is the main component of polysaccharides; sugar quantification was conducted to estimate the bacterial EPS production. The results showed that isolate YNA59 produced a significant amount of sucrose (0.51± 0.12 mg/ml) compared to the control (0.07 ± 0.003 mg/ml) (Fig. 2B).

### Isolate YNA59 Oxidative Stress Tolerance

**Fig. 3:**
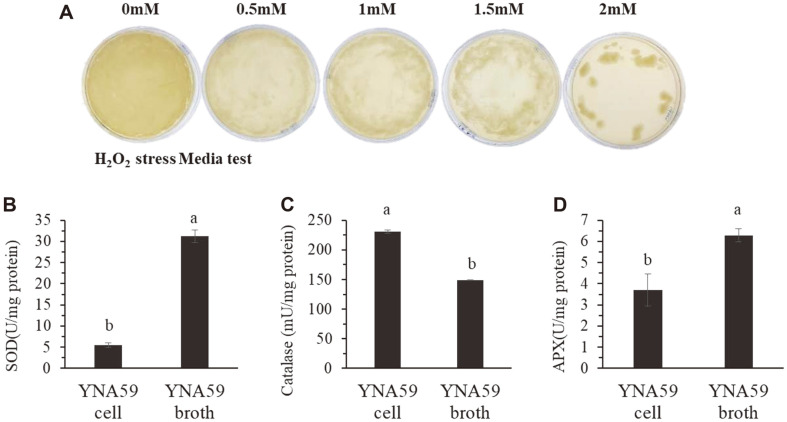
Isolate YNA59 tolerance to oxidative stress. (A) showed resistance to H_2_O_2_, (B) produce significant amount of superoxide dismutase SOD activity, (C) Catalase activities (CAT) and (D) Ascorbic peroxidase (APX) in the YNA59 broth (culture filtrate) and YNA59 cell (pellets were mix in distilled water). Each data point is the mean of at least three replicates. Error bars represent standard errors. The bars presented with different letters are significantly different from each other as evaluated by DMRT.

Isolate YNA59 could tolerate high oxidative stress and grow efficiently on LB agar plates containing up to 2 mM H_2_O_2_ (Fig. 3A). Similarly cultures broth analysis of isolate YNA59 indicates significantly higher SOD, CAT, and APX activities, demonstrating the presence of highly active antioxidant mechanism (Figs. 3B-3D).

### Bacterial Isolate YNA59 Regulates Broccoli Growth under Drought Stress

**Fig. 4:**
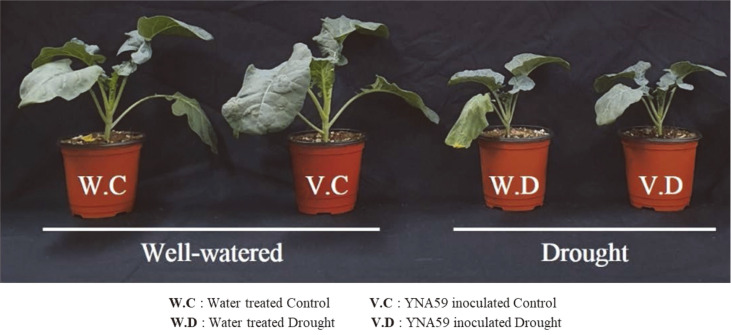
Effects of selected bacterial isolate YNA59 on the growth of broccoli plants under normal and drought stress.

Drought stress adversely affected the growth attributes of broccoli plants. However, isolate YNA59 significantly enhanced drought stress tolerance by regulating plant growth, plant biomass, and other biochemical attributes after 14 days of inoculation. Under drought stress, a decrease in plant height (30%), leaf length (40.17%), leaf width (34.73%), fresh weight (61.23%), and chlorophyll content (51.28%) were observed. However, isolate YNA59 induced significant increase in plant height (12.14%), leaf length (10%), leaf width (14.51%), fresh weight (23.18%), and chlorophyll content (61.78%) in drought stressed plants compared to the control (Fig. 4, Table 1).

Soil moisture content was also significantly higher in the YNA59-inoculated pots than the non-inoculated pots. The final soil moisture was measured 14 days after transfer, which showed that YNA59 treatment retained more soil moisture in drought stress pots (61%) compared to the control (16.9%) (Table 1).

### Effect of Isolate YNA59 on Plant Endogenous Phytohormones

**Fig. 5:**
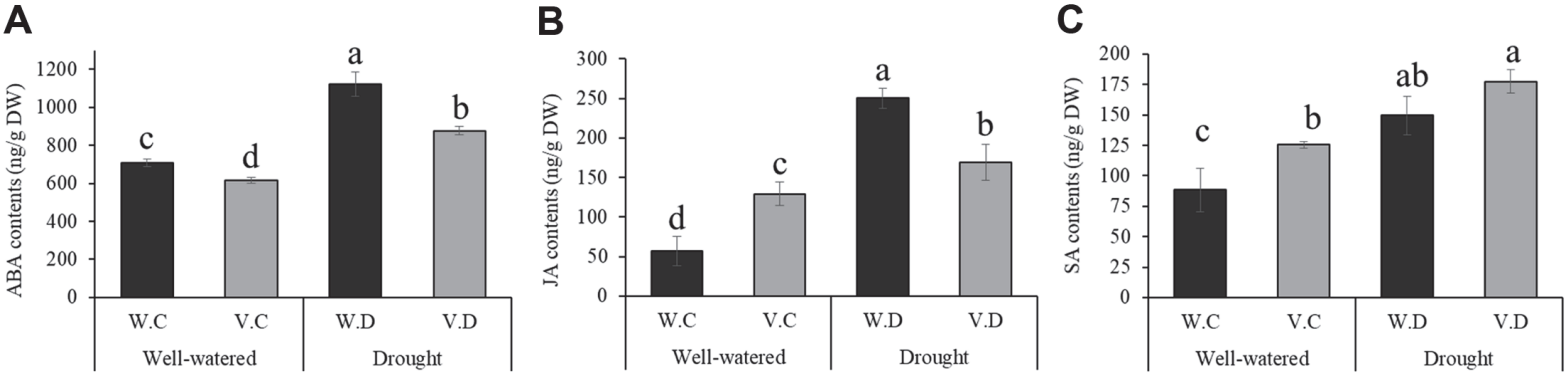
Endogenous abscisic acid (ABA), jasmonic acid (JA), and salicylic acid (SA) quantification in broccoli plants inoculated with YUNA59. (**A**) Demonstrates ABA, (**B**) JA, and (**C**) shows the amount of SA under normal and drought stress. Each data point is the mean of at least three replicates. Error bars represent standard errors. The bars presented with different letters are significantly different from each other as evaluated by DMRT.

Drought stress induced a significant increase in ABA (1.57 folds) and JA (5 folds) contents of broccoli plants (Figs. 5A and 5B). In isolate YNA59 inoculated broccoli, we observed a decrease in ABA (0.77 folds) and JA (0.7 folds) contents (Figs. 5A and 5B). However, in contrast to endogenous ABA and JA levels, increase of 20% in the endogenous SA contents was observed in isolate YNA59-inoculated broccoli plants (Fig. 5C).

### DAB Staining and Antioxidant Quantification in Broccoli

**Fig. 6:**
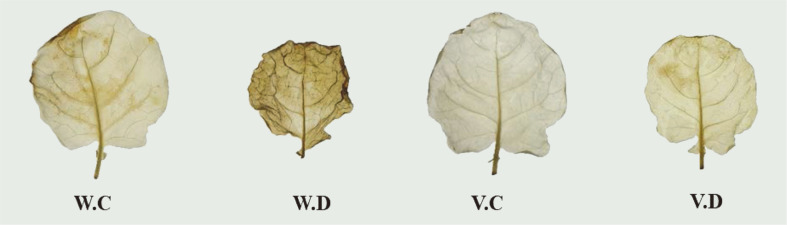
Detection of hydrogen peroxide in broccoli leaves under normal and drought stress using 3,3’-diaminobenzidine (DAB) staining method.

Drought stresses leads to the generation of ROS *i.e.*, H_2_O_2_, thereby causing oxidative damage to cells. DAB staining results showed more reddish-brown color development in drought stress control broccoli leaves than isolate YNA59-inoculated leaves. Thus, it was speculated that YNA59 treatment would help maintain the ROS equilibrium by minimizing excessive H_2_O_2_ accumulation in plant leaves (Fig. 6).

**Fig. 7:**
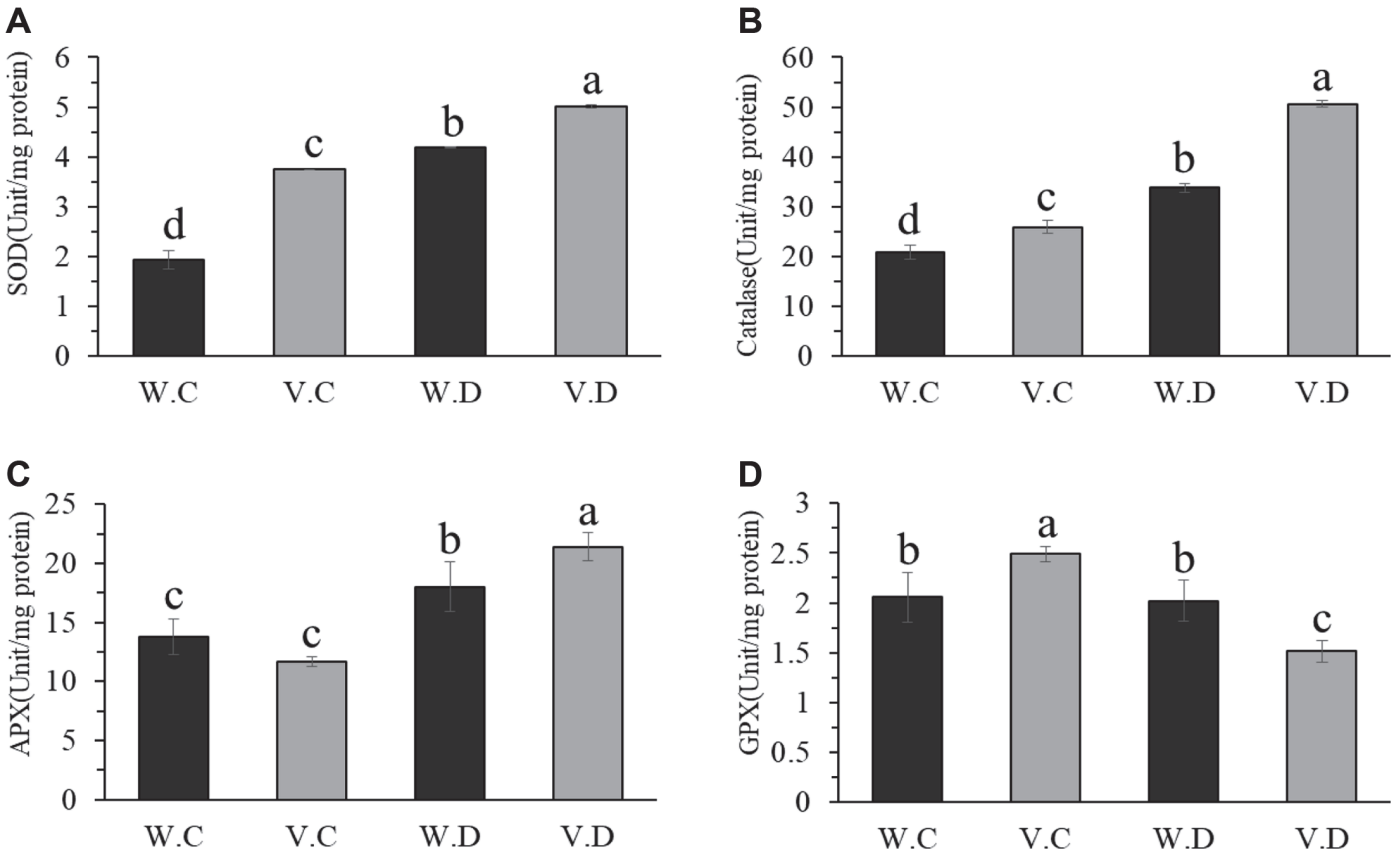
The effect of bacterial isolates YNA59 on different antioxidants. (**A**) Superoxide dismutase (SOD); (**B**) Catalase (CAT); (**C**) Ascorbic peroxidase (APX), and (D) Guaiacol peroxidase (GPX) contents in broccoli plants under normal and drought stress. Each data point is the mean of three replicates. Error bars represent standard errors. The bars presented with different letters are significantly different from each other as evaluated by DMRT.

Excessive accumulation of ROS in broccoli was eliminated through chemical reactions of enzymatic antioxidant chemicals such as SOD, CAT, and APX and non-enzymatic antioxidant chemicals such as glutathione, ascorbate, and polyphenol. This study was conducted to investigate the changes in antioxidant enzymes in broccoli during drought. SOD results showed that an increase in SOD content was observed under drought stress, however a significant increase in SOD content in isolate YNA59 treated broccoli plants were observed compared with drought stress and control plants (Fig. 7A). A similar trend was observed in the CAT and APX enzymatic activity, which showed higher proportions in drought stress broccoli plants inoculated with isolate YNA59 (Figs. 7 B and C). In contrast to SOD, CAT, and APX, GPX activity showed a significantly decreased value compared to drought stress and control plants (Fig. 7D).

### Identification of Bacterial Isolate YNA59

**Fig. 8:**
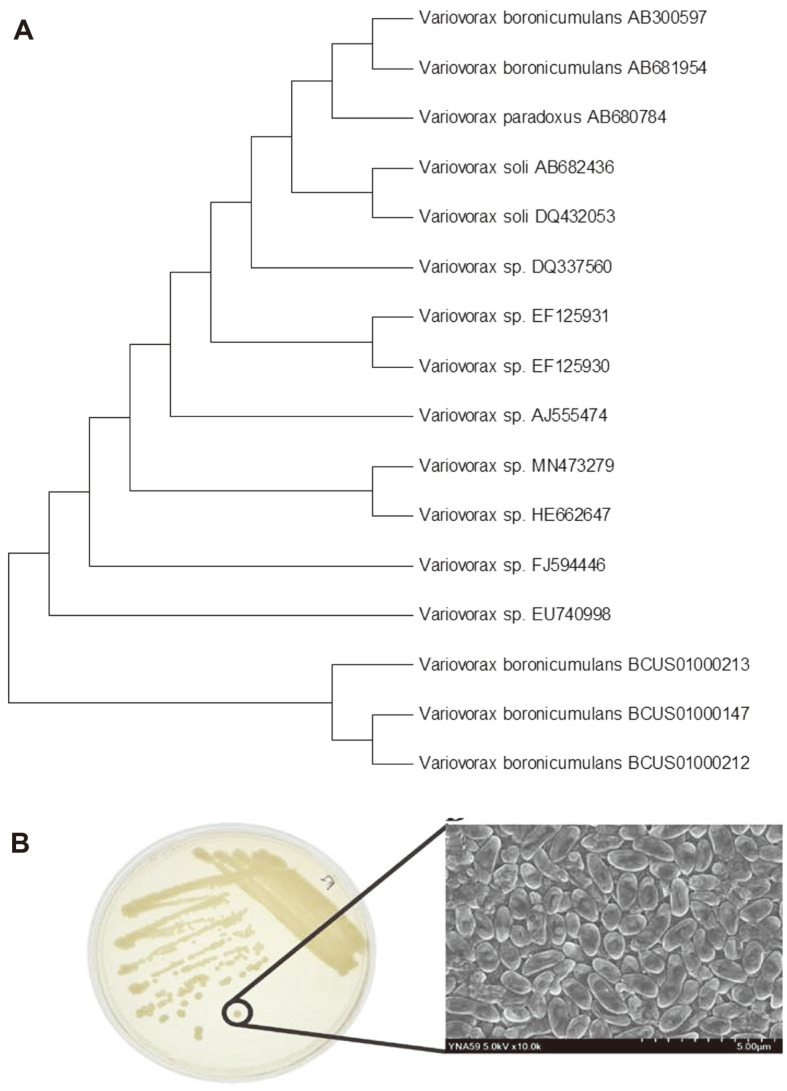
Phylogenetic tree of YNA59 was constructed using 16S rRNA sequences by neighbor joining (NJ) and maximum likelihood methods. (**A**) Phylogenetic tree of YNA59 was constructed using 16S rRNA sequences by neighbor joining (NJ) and maximum likelihood methods; (**B**) Microscopic observation of YNA59 using a scanning electron microscope.

Molecular identification and phylogenetic analysis of isolate YNA59 were performed by amplifying and sequencing the 16S rRNA after which it was compared to the database of known 16S rRNA gene sequences and BLAST search tool of NCBI data base/EzTaxon. Our results revealed that isolate YNA59 exhibited a high sequence identity (99%) with Variovorax sp. and sequence was submitted to NCBI with GenBank accession no MN473279. Furthermore, the morphological characteristic of isolate YNA59 was investigated using field emission scanning electron microscopes (FE-SEM). Variovorax sp. YNA59 colony, which grew in LB broth was yellowish, smooth, and glistening (Fig. 8A). Microscopic observation of Variovorax sp. YNA59 displayed a curved rod-shaped, occurring as either single or pairs of cells and approximately 0.61 × 2.04 μm long (Fig. 8B).

## Discussion

The robust nutritional properties and different beneficial characteristics of broccoli have contributed to its increased demand worldwide. There is a need to boost the production of broccoli under drought conditions worldwide. To ease the adverse effects of drought stress, various methods including plant breeding, chemical fertilizer, tissue culture and genetic engineering have been used [19-23]. Similarly, several researchers have suggested the usage of drought tolerant microbes as a promising alternative to alleviate plant stress caused by drought stress. Recently the role of microbes in the management of drought stressor has begun gaining importance in wheat [41], pepper [42, 43], tomato [44], Chinese liquorice [45], Arabidopsis [46], timothy [47], and maize [48].

Broccoli, an important cultivated vegetable is sensitive to drought stress as its growth, yield, and quality are highly affected under this condition [49, 50]. The decline in growth attributes under drought stress is because it directly affects photosynthesis and transpiration process [50]. Drought stress induces numerous physiological changes, such as abnormal metabolic processes, stomatal conductance, and hormone biosynthesis, which result in a disturbance of the normal growth and development [51, 52]. In this study, our results revealed that drought stress inhibits plant growth and development; however, inoculation with the drought tolerant isolate YNA59 markedly alleviated the adverse effect of drought stress (Fig. 4, Table 1). The beneficial effects of selected bacterial isolate YNA59 on broccoli plants were likely due to plant growth-promoting traits such as PEG tolerance, EPS formation, and phytohormone ABA (Figs. 1 and 2). Isolate YNA59 have the ability of EPS formation and it was reported that EPS producing bacteria are able to maintain higher water potential around the roots and in soil, thus protecting the plants against drought stress [53, 54].

Among other biochemical and physiological process of plants, photosynthesis is one of the most sensitive to abiotic stresses, including drought stress, that adversely affect chloroplast function and stomatal regulation [55, 56]. Plants have evolved several mechanisms to relieve drought stress, such as accumulation of osmolytes, reprogramming of phytohormone production, stomatal closure, and enhanced antioxidant defense system [55, 56]. Similarly, the chlorophyll results of our current study showed that a decrease in chlorophyll content was observed when the plants were subjected to drought stress; however, inoculation with YNA59 mitigated drought stress and increased the chlorophyll contents (Table 1). This increase in the chlorophyll content may also be explained by the increased photosynthetic leaf area (length and width) of plants inoculated with bacteria compared with that of non-inoculated plants, which was reduced due to drought stress (Table 1). Similar results were also reported in previous studies showing that the use of drought stress tolerant bacteria could increase the total chlorophyll content in basil, Chinese liquorice, maize, and rice plants under drought stress [45,57-59].

Phytohormones play a vital role in plant by regulating plant growth and stress tolerance to maintain the viability of the plants under stress conditions. Studies have demonstrated that among other phytohormones, abscisic acid is actively involved in the response to drought stress. Under stress conditions, plants regulate stress hormones such as ABA through active chemical signals, which contribute to stomatal closure and water conservation [60, 61]. Plant-microbe interaction has been previously reported that mitigate the adverse effects of abiotic stress in plants through reducing ABA levels [30, 62, 63]. The results of our current study showed that the inoculation of isolate YNA59 enhance plant growth parameters and mitigate drought stress through the reduction of ABA accumulation (Fig. 5A). Similarly, SA and JA is another phyto-hormone that plays an important role in abiotic stress tolerance [64]. These phyto-hormones stimulate signal molecules and play anti-oxidative role to protect the plants from oxidative stress and ROS generation [65, 66]. Previously, in various plants, including Kentucky blue grass, grapevine, potato, grape, *Arabidopsis*, bean, and tomato; the accumulation of SA has been associated with drought stress tolerance [67-71]. In this study, higher endogenous SA levels were observed in isolate YNA59-inoculated broccoli plants under normal and drought stress (Fig. 5C). Our findings also support previous studies, which revealed that bacterial inocula enhances endogenous SA levels and decreases that of JA and enhances the growth attributes of plants under abiotic stress [24, 28, 31, 72]. Furthermore, drought stress increases the accumulation of ROS (singlet oxygen, superoxide anion, and H_2_O_2_), which cause cellular toxicity and lipid peroxidation along with protein degradation [49]. However, plants can reduce the damaging effect of ROS by the development of its antioxidant system consisting of glutathione reductase (GR), SOD, MDA, and APX, which can protect the plants against cellular stress, scavenge excess ROS and remove free radicals. The first antioxidant produced is SOD, which converts O_2_•- radicals to H_2_O_2_. This H_2_O_2_ is further detoxified by CAT and APX into water and oxygen [43]. Analysis of culture broth of isolate YNA59 indicates a significant amount of SOD, CAT, and APX activities (Fig. 3). Similarly, broccoli plants inoculated with YNA59 produced less ROS and exhibited increased antioxidant SOD, CAT, and APX activity while, GPX peroxidase activity showed opposed aspect with APX under drought stress (Fig. 7). Similar results were reported with the inoculation of drought tolerant bacteria, which significantly increased the activity of different ROS-scavenging enzymes in maize, basil, and rice [14,57-59].

## Conclusion

Our current findings showed that drought tolerant rhizospheric isolate Variovorax sp. YNA59 is capable of producing different PGP traits, such as catalase assay, EPS formation, and PEG tolerance. Similarly, isolate YNA59 greatly enhanced the growth attributes, leaf width and length, chlorophyll content, and soil moisture content of broccoli plants. This improvement in plant growth was induced by isolate YNA59, which regulated plant endogenous phytohormones (ABA, JA, and SA) and antioxidants (APX, SOD, CAT, and GPX), as severe drought stress leads to excessive ROS, causing membrane and protein degradation. Based on the ameliorative qualities of isolate YNA59, it can be used for mitigating drought stress and as an ecofriendly bio-fertilizer for sustainable agriculture in drought-affected areas of the world.

## Supplemental Materials



Supplementary data for this paper are available on-line only at http://jmb.or.kr.

## Figures and Tables

**Table 1:** Effects of selected bacterial isolate YNA59 on the growth of broccoli plants under normal and drought stress.

Treatment	Height (cm)	Leaf length (cm)	Leaf width (cm)	Fresh weight (g)	Soil moisture (%)	Chlorophyll content (CC)
W.C	20.0 ± 1.1a	11.7 ± 0.5a	9.5 ± 0.4b	17.8 ± 0.8b	70.0 ± 3.9b	554.22 ± 40c
V.C	21.8 ± 0.7a	12.1 ± 0.7a	10.4 ± 0.6a	20.3 ± 0.7a	81.8 ± 3.7a	572.8 ± 28b
W.D	14.0 ± 0.5b	7.0 ± 0.7b	6.2 ± 0.6c	6.9 ± 0.7d	34.1 ± 6.0d	531.57 ± 34d
V.D	15.7 ± 0.7b	7.7 ± 0.7b	7.1 ± 0.6c	8.5 ± 0.7c	55.0 ± 7.3c	588.74 ± 35a

W.C: Control with well-watered treatment, V.C: YNA59 inoculation well-watered treatment, W.D: Control drought stress, V.D: YNA59 inoculation with drought stress. Each data point is the mean of three replicates. Error bars represent standard errors. The bars presented with different letters are significantly different from each other as evaluated by DMRT analysis .
