# Supplementary material for: Enhancement of Drought-Stress Tolerance of Brassica oleracea var. italica L. by Newly Isolated Variovorax sp. YNA59
Source: J Microbiol Biotechnol. 2020 Aug 13;30(10):1500–9. doi: 10.4014/jmb.2006.06010 (PMC9728237; doi:10.4014/jmb.2006.06010)

J. Microbiol. Biotechnol.
https://doi.org/10.4014/jmb.jmb.2006.06010

S. Figure 1

Screening isolates for Catalase and exopolysaccharide (EPS) activity on orange media and Congo [illegible]dium are shown. (A) Shows the capability of catalase production, (B) EPS production, and (C) [illegible] of isolate YNA59 under with different concentration of PEG6000.

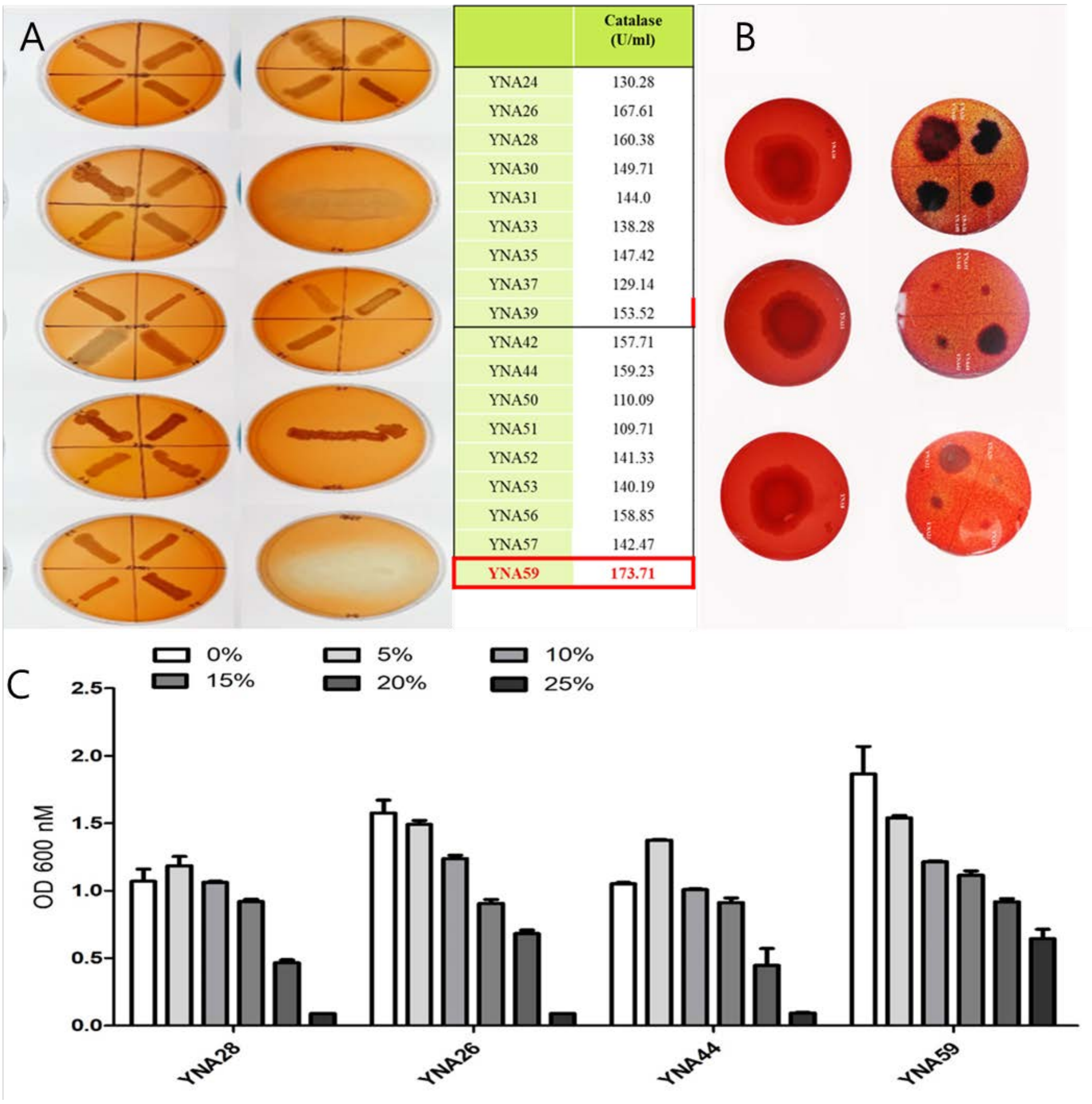

Supplement: Supplementary file 1 [file JMB-30-10-1500-supple.pdf]
